# A Fatal Case of 3-Hydroxyisobutyryl-CoA Hydrolase Deficiency in a Term Infant with Severe High Anion Gap Acidosis and Review of the Literature

**DOI:** 10.1155/2024/8099373

**Published:** 2024-06-30

**Authors:** Surasak Puvabanditsin, Ian Lee, Natasha Cordero, Keisha Target, Su Young Park, Rajeev Mehta

**Affiliations:** Rutgers Robert Wood Johnson Medical School, New Brunswick, New Jersey, USA

## Abstract

3-hydroxy isobutyl-CoA hydrolase (HIBCH) deficiency is a recently described, rare inborn error of valine metabolism associated with a Leigh syndrome-like phenotype, neurodegenerative symptoms, and caused by recessive mutations in the HIBCH gene. We report the most severe case to date of an intrauterine growth-restricted term male who presented with severe acidosis and a high anion gap soon after birth. The manifestation was fatal that led to death within 36 hours of life. The diagnosis was made postnatally by Whole Genome Sequencing (WGS). We report a rapid and fatal event of HIBCN in a neonate and review of the literature.

## 1. Introduction

3-hydroxy isobutyl-CoA hydrolase (HIBCH) deficiency (MIM: #250620) is a rare inborn error of valine metabolism characterized by neurodegenerative symptoms such as developmental delay, regression, hypotonia, dystonia, ataxia, encephalopathy, and feeding difficulties (PMID: 17160907, 24299452, 37604814, 36200804, 29703962) [1–3]. HIBCH deficiency is caused by a defect in the HIBCH enzyme, leading to a deficiency in the conversion of 3-hydroxy-isobutyryl-CoA to 3-hydroxy-isobutyric acid, a critical step in valine catabolism [4, 5]. Since its introduction in 1982, only a few cases have been reported [6, 7]. Presentation is typically in early infancy or within the first year of life. It is caused by recessive mutations in the HIBCH gene. Laboratory findings include increased plasma 4 hydroxybutyrylcarnitine levels and increased lactic acid (PMDPMID: 33506479) [2, 8]. Our patient was a term male who presented with severe metabolic acidosis and a high anion gap soon after birth and is perhaps the most severe case reported to date.

## 2. Case Report

This is a 1785-g male neonate who was born at 37 weeks of gestation to a 37-year-old gravida 3 para 2012 mother by scheduled cesarean section because of severe intrauterine growth restriction (IUGR). Apgar scores were 3 and 7 at 1 and 5 minutes, respectively. Prenatal care was complicated with a vanishing twin and IUGR. Amniocentesis was performed and no major chromosomal abnormality was noted. The family history was unremarkable and there is no consanguinity. Birth weight was 1785 g (<3^rd^ centile), length 46 cm (<3^rd^ centile), and head circumference was 30.5 cm (<3^rd^ centile). The physical findings at birth included a small for gestational age infant with dysmorphic facies and an enlarged anterior fontanelle. The infant was hypotonic and inactive with respiratory distress and was admitted to NICU and placed on nasal CPAP. Initial blood gas at 2 hours of age showed pH 7.21, pCO_2_ 27 mmHg, pO_2_ 92 mmHg, base deficit −15 milliequivalents(meg)/liter (L). He was intubated and placed on a conventional ventilator, and an infusion of sodium bicarbonate 2 meq per kilogram body weight was administered. Arterial blood gas at 6 hours of life showed pH 6.96, pCO_2_ 47 mmHg, pO_2_ 75 mmHg, and base deficit −21 meq/L. The patient remained hypotonic, lethargic, and unresponsive. The initial serum glucose was 10 mg/dL. Intravenous 10% dextrose boluses were administered, and continuous intravenous glucose was begun. Serum electrolytes were Na 139 meq/L, K 5 meq/L, Cl 98 meq/L, bicarbonate (HCO_3_) 9 meq/L, anion gap 32 meq/L, and Ca 8.5 mg/dL; high serum lactate of 17 mmol/L was also noted. Liver function test revealed AST 100 U/L, ALT 21 U/L, ALK Phos 232 U/L, total protein 4.3 g/L, albumin 2.8 g/L, total bilirubin 3.9 mg/dL, and direct bilirubin 0.3 mg/dL. Serum ammonia was 595. Intravenous infusion of 2.5 mg/kilogram body weight of ammonal (sodium phenylacetate and sodium benzoate) was begun at 24 hours of life. While awaiting hemodialysis, the patient's condition deteriorated, and he expired at 34 hours of age. An autopsy was refused.

## 3. Cytogenetic and Molecular Studies

Whole genome sequencing (WGS) was performed using next-generation sequencing (NGS) technology. PCR-free library preparation was performed before WGS. The test assesses single nucleotide variants (SNVs), small deletions and insertions, larger deletions and duplications, the mitochondrial genome, SMN1 and SMN2 copy number analysis, and repeat expansions in PHOX2B and DMPK. Alignment and variant calling were performed with the Illumine DRAGEN pipeline using the official reference build 37.1 (hg19). Cony number variation (CNV) calling was performed using a combination of CNV callers. Repeat expansion calling for PHOX2B and DMPK was performed using Expansion Hunter (v.4.0.3) in DRAGEN 3.10.4. A heterozygous c.129dup (p.GLy44ArgfsTer20) variant and heterozygous c.830T > A (p.Val277Glu) variant of the HIBCH gene were detected in the patient. Analysis of the parental samples revealed that the father is heterozygous for the c.129dup (p.GLy44ArgfsTer20) variant and the mother is heterozygous for the c.830T > A (p.Val277Glu) variant. Based on the available evidence, c.129dup (p.GLy44ArgfsTer20) is classified as likely pathogenic and c.830T > A (p.Val277Glu) is classified as a variant of uncertain significance [1, 9–15].

## 4. Discussion

**Figure 1:**
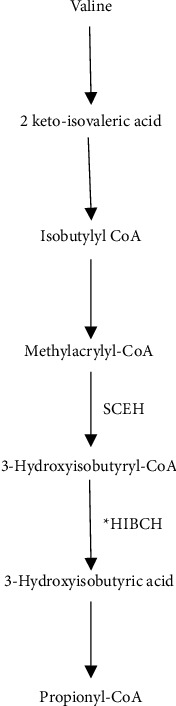
The valine metabolism, highlighting the role of 3-hydroxyisobutyryl-Co A hydrolase (HIBCH) and short-chain enoyl-CoA (SCEH).

The HIBCH gene located on chromosome 2q32.2 encodes for the mitochondrial enzyme 3-hydroxyisobutyryl-CoA hydrolase that catalyzes the conversion of 3-hydroxyisobutyryl-CoA to 3-hydroxyisobutyrate (Figure 1), the fifth step in the catabolism of valine (PMID: 8824301) [13]. Valine is an essential branched-chain amino acid (BCAA) that is present in all protein-containing foods. Valine is metabolized in the mitochondria via the enzymatic pathway that includes HIBCH [14]. In the initial step, valine converts to methacrylyl-CoA and then to 3-hydroxyisobutyryl-CoA. In the next step, HIBCH converts 3-hydroxyisobutyryl-CoA to 3-hydroxyisovaleric acid and finally to the product propionyl Co-A [16].

The pathogenesis of HIBCH deficiency can be explained through the buildup of 3-hydroxy-isobutyryl-CoA, which fluxes upward through the reversible crotonase enzyme. This leads to the intracellular accumulation of methacrylyl-CoA, a proximal metabolite in the catabolism of valine. The methacrylyl-CoA accumulates in the mitochondria causing secondary mitochondriopathy by reacting with mitochondrial enzymes containing essential cysteine residues including PDHC and respiratory chain enzymes producing irreversible binding cofactors, such as CoA and lipoic acid [4–6, 14, 15, 17]. A lack of CoA inhibits the Krebs cycle and results in a reduction of adenosine triphosphate (ATP) production. Due to the functional defect in the mitochondrial Electron Transport Chain, the cells must switch from respiration to glycolysis to compensate for the ATP deficit resulting from the mitochondrial dysfunction. This results in an increase in lactate levels [3].

The mutations of genes encoding mitochondrial proteins required for the assembly and function of electron transport chain (ETC) complexes, as well as the use of certain drugs and other physiological stress conditions have all been associated with the onset of lactic acidosis (LA) [18]. LA is characterized by the buildup of lactate due to decreased mitochondrial respiration, which can lead to the acidification of tissues.

Pathogenic variations in the HIBCH gene are associated with an autosomal recessive HIBCH deficiency (MIM: #250620). Features include dysmorphic facies, nystagmus, strabismus, seizures, myoclonus, and paroxysmal dyskinesia (PMID: 33506479). The reported neuroimaging abnormalities include T2 hyperintensity in the basal ganglia, progressive brain atrophy, optic nerve atrophy, and brainstem lesions [9]. The nonspecific findings in the reported cases of HIBCH deficiency could have overlapping similarities with the clinical manifestations and neuroimages of Leigh syndrome or other mitochondrial diseases.

To date, 28 HIBCH deficiency cases from 15 families have been reported in the worldwide literature (Table 1) [3–5, 8, 12, 15, 19–27]. The first case of HIBCH deficiency was reported in 1982 by Brown et al. in a male infant with multiple physical malformations: dysmorphic facial features, multiple vertebral anomalies, tetralogy of Fallot, and agenesis of the cingulate gyrus, and corpus callosum, failure to thrive, and marked hypotonia [6] and was born to parents who were cousins. The second case of HIBCH deficiency was also a male infant but it was the first reported case of a baby born to nonconsanguineous parents and presenting with ataxia, dysmetria, tremors, developmental delay, and metabolic acidosis. It is pertinent to mention here that metabolic acidosis was also seen in our patient. The third reported case was the 4-year-old son of nonconsanguineous parents from Iran, who presented with ataxia, dysmetria, general hypotonia, developmental delay, and hyperactive deep tendon reflexes, with inappropriate walking, talking, and growth parameters, and recurrent attacks of symptoms such as weakness, myoclonus, and eye nystagmus following febrile illnesses. The brain MRI of the boy showed bilateral high-signal lesions in the globus pallidus (Leigh-like syndrome). Although consanguinity was reported in some of the reported cases, our patient was not from a consanguineous relationship. Given the clinical variability in its presentation, several differential diagnoses and its similarities with Leigh syndrome, which is a common neurometabolic disorder associated with different genes, the prevalence of HIBCH deficiency are probably underestimated [3, 26].

Next-generation sequencing approaches are an effective tool for identifying the underlying genetic basis in patients suspected of mitochondrial disorders. Rapid whole exome sequencing (WES) demonstrated a heterozygous pathogenic c.129dup (p.Gly44ArgfsTer20) variant of the HIBCH gene in our patient with a paternal origin. This frameshifting variant in exon 3 of 14 is predicted to result in the loss of normal protein function through a compound protein truncation or nonsense-mediated mRNA decay. Loss-of-function variation in HIBCH is an established mechanism of disease (PMID: 29703962). This variant has been previously reported as a compound heterozygous change in patients with 3-hydroxy isobutyl-CoA hydrolase deficiency (PMID: 26163321). This variant has been reported in the ClinVar database (Variation ID: 208531). The c.129dup (p.Gly44ArgfsTer20) variant is present in the heterozygous state in the Genome Aggregation Database (gnomAD) population database at a frequency of 0.001% (4/250434) and is absent in the homozygous state; it is presumed to be very rare. This case demonstrates the importance of rapid WES and follow-up functional testing in establishing a diagnosis when metabolic disease is suspected but lacks an expected biochemical signature. Thus, HIBCH deficiency must be kept in mind in any neonates with high anion gap metabolic acidosis.

In conclusion, we have described a fatal manifestation of HIBCH deficiency, a disorder of valine catabolism, in a term newborn with intrauterine growth retardation. Due to the rarity of the occurrence of this disorder of an inborn error of metabolism, it is important to evaluate properly and pay attention to the diagnostic clue of high anion gap metabolic acidosis.

## Figures and Tables

**Table 1:** HIBCH deficiency cases reported in the literature.

References	Number of case	Gender	Ethnicity	Consanguinity	HIBCH gene mutation	Clinic findings
[6]	1	M	Egypt	+	Lys74Leufs*∗*13	Hypotonia

[15]	1	M	ND	−	Tyr122Cys/IVS2-3C > G	Hypotonia
		M				

[5]	2	M	Pakistan	+	c.950G < A (p.Gly317Glu)	Hypotonia
		M				

[24]	2	F	Japanese	−	Ala96Asp	Hypotonia
		F				

[26]	1	M	Tunesia	+	p.Lys377	Hypotonia

[23]	1	F	Chinese	−	c.1027C > G	Development delay
					c.79-1G > T	

[27]	1	F	Caucasian	−	c.517 + 1G > A	Hypotonia
					c.410C > T (p.A137V)	

[4]	2	M	Lebanese	−	c.196C > T (p.Arg66Trp)	Hypotonia
		F				

[19]	3	M	Turkish	+	c.913A > G	Hypotonia
		F				
		M				

[19]	2	M	Turkish	+	c.913A > G	Dystonia
		M				

[22]	1	F	Chinese	−	c.1027C > *G* (p.H343D)	Dystonia

[8]	1	M	Chinese	−	c.439-2A > G	Dystonia

[12]	1	M	Chinese	−	c.304 + 3A > G	Hypotonia

[25]	1	M	Iran	−	c.641C > T (p.Thr214Ile)	Hypotonia
					c.913A > *G* (p.Thr305Ala)	

[3]	2	F	Columbia	−	c.808A > *G* (p.Ser270Gly)	Hypotonia
		M				

[21]		F	ND	−	ient	Hypotonia
					(c.488G > T, p.C163F)	

[20]	5	F	Turkish	+	c.452C > T	Development delay
		F			p.Ser151Leu	
		M				
		F				
		M				

Our patient		M	Pakistan	−	c.129dup (p.Gly44ArgfsTer20)	Severe metabolic acidosis
					c.830T > A (p.Val277Glu)	

ND: no definition.

## Data Availability

The data that support the findings of this study are available from the corresponding author upon reasonable request.
