# Corrigendum to “A Fatal Case of 3-Hydroxyisobutyryl-CoA Hydrolase Deficiency in a Term Infant With Severe High Anion Gap Acidosis and Review of the Literature”

**DOI:** 10.1155/crig/9827627

**Published:** 2025-07-25

S. Puvabanditsin, I. Lee, N. Cordero, K. Target, S. Y. Park, R. Mehta, “A Fatal Case of 3-Hydroxyisobutyryl-CoA Hydrolase Deficiency in a Term Infant With Severe High Anion Gap Acidosis and Review of the Literature,” *Case Reports in Genetics*, 2024, https://doi.org/10.1155/2024/8099373.

In the article, there is an error in Table 1, whereby the ethnicity of the reported patient of this case report in the final row is incorrect. The ethnicity should be India, not Pakistan. The correct Table 1 is shown below:

We apologize for this error.

## Figures and Tables

**Table 1:** HIBCH deficiency cases reported in the literature.

References	Number of cases	Gender	Ethnicity	Consanguinity	HIBCH gene mutation	Clinic findings
[6]	1	M	Egyptian	+	Lys74Leufs × 13	Hypotonia
[15]	1	M	ND	–	Tyr122Cys/IVS2-3C > G	Hypotonia
		M				
[5]	2	M	Pakistani	+	c.950G < A (p.Gly317Glu)	Hypotonia
		M				
[24]	2	F	Japanese	–	Ala96Asp	Hypotonia
		F				
[26]	1	M	Tunisia	+	p.Lys377	Hypotonia
[23]	1	F	Chinese	–	c.1027C > G	Development delay
					c.79-1G > T	
[27]	1	F	Caucasian	–	c.517 + 1G > A	Hypotonia
					c.410C > T (p.A137V)	
[4]	2	M	Lebanese	–	c.196C > T (p.Arg66Trp)	Hypotonia
		F				
[19]	3	M	Turkish	+	c.913A > G	Hypotonia
		F				
		M				
[19]	2	M	Turkish	+	c.913A > G	Dystonia
		M				
[22]	1	F	Chinese	–	c.1027C > G (p.H343D)	Dystonia
[8]	1	M	Chinese	–	c.439-2A > G	Dystonia
[12]	1	M	Chinese	–	c.304 + 3A > G	Hypotonia
[25]	1	M	Iranian	–	c.641C > T (p.Thr214Ile)	Hypotonia
					c.913A > G (p.Thr305Ala)	
[3]	2	F	Columbian	–	c.808A > G (p.Ser270Gly)	Hypotonia
		M				
[21]		F	ND	–	ient	Hypotonia
					(c.488G > T, p.C163F)	
[20]	5	F	Turkish	+	c.452C > T	Development delay
		F			p.Ser151Leu	
		M				
		F				
		M				
Our patient		M	Indian	–	c.129dup(p.Gly44ArgfsTer20)	Severe metabolic acidosis
					c.830T > A(p.Val277Glu)	

Abbreviation: ND, no definition.

